# Genetic polymorphism of HLA-DRB1 alleles in Mexican mestizo patients with abdominal aortic aneurysms

**DOI:** 10.1186/s12881-019-0833-8

**Published:** 2019-06-07

**Authors:** Javier E. Anaya-Ayala, Susana Hernandez-Doño, Monica Escamilla-Tilch, Jose Marquez-Garcia, Kemberly Hernandez-Sotelo, Rodrigo Lozano-Corona, Daniela Ruiz-Gomez, Julio Granados, Carlos A. Hinojosa

**Affiliations:** 1 Department of Surgery, Section of Vascular Surgery and Endovascular Therapy, Instituto Nacional de Ciencias Medicas y Nutricion Salvador Zubirán, Vasco De Quiroga 15, Tlalpan, Sección XVI, 14080 México City, Mexico; 2 Universidad Nacional Autonoma de Mexico, Faculty of Medicine, Division for Postgraduate Studies, Master and Doctoral Degree Program, Mexico City, Mexico; 3 Department of Transplant Surgery, Division of Immunogenetics, Instituto Nacional de Ciencias Medicas y Nutricion Salvador Zubirán, Mexico City, Mexico; 4 Centro Medico Nacional 20 de Noviembre, Biochemistry Unit, Mexico City, Mexico; 5 Instituto Nacional de Enfermedades Respiratorias Ismael Cosio Villegas, Biochemistry Unit, Mexico City, Mexico

**Keywords:** Abdominal aortic aneurysms, HLA-DRB1, Genetic risk, Susceptibility, Mexican population

## Abstract

**Background:**

Multiple factors are implicated in the etiology and pathogenesis of Abdominal Aortic Aneurysms (AAA). Available literature of genetic studies has previously suggested the possible roles of autoimmunity, genetic predisposition and ethnic susceptibility. Due to the association with autoimmune diseases and proven application in population genetics, we aimed to investigate alleles of the Class II Human Leukocyte Antigens (HLA-DRB1) in the Mexican Mestizo population with aortic aneurysms and determine possible associations with susceptibility.

**Methods:**

We performed a case Control Study; the HLA molecular typing was completed for DRB1 loci by LabType Sequence-Specific Oligonucleotide (SSO) SSO-OneLambda kit (Applied Biosystems; Thermo Fisher Scientific. Inc.) in the studied individuals. Allele frequencies (af) were determined, associations were assessed by chi square or fisher exact tests at significance level (< 0.05), and Odds Ratios (OR) were calculated using the STATA software version 14.

**Results:**

The genetic polymorphism of HLA-DRB1 of fifty one patients (70% males with a mean age of 71 years) with atherosclerotic or also known as degenerative AAA were compared with 99 unrelated patients (60% males, mean age 65 years) without the disease [Control group (CG)] from the same ethnic group. We examined a total of 102 Class II HLA-DRB1 alleles of AAA patients and 198 from CG. When comparing af, we observed the HLA-DRB1*01 af of 0.139 in the AAA compared to 0.05 in the CG [*p* = 0.015, OR 3, 95% confidence interval (CI) 1.29–7.08], the HLA-DRB1*16 af were 0.109 in the AAA and 0.025 in CG (*p* = 0.006, OR 4.7, 95% CI 1.59–13.98).

**Conclusions:**

Our study confirmed increased frequencies of the alleles HLA-DRB1*01 and HLA-DRB1*16 and their association to the development of AAA in Mexican Mestizo patients. The utility of genetic testing may assist in identifying individuals at genetic risk for the development of this disease in different ethnic groups, who might benefit from earlier ultrasound screening and closer imaging surveillance.

## Background

**Fig. 1:**
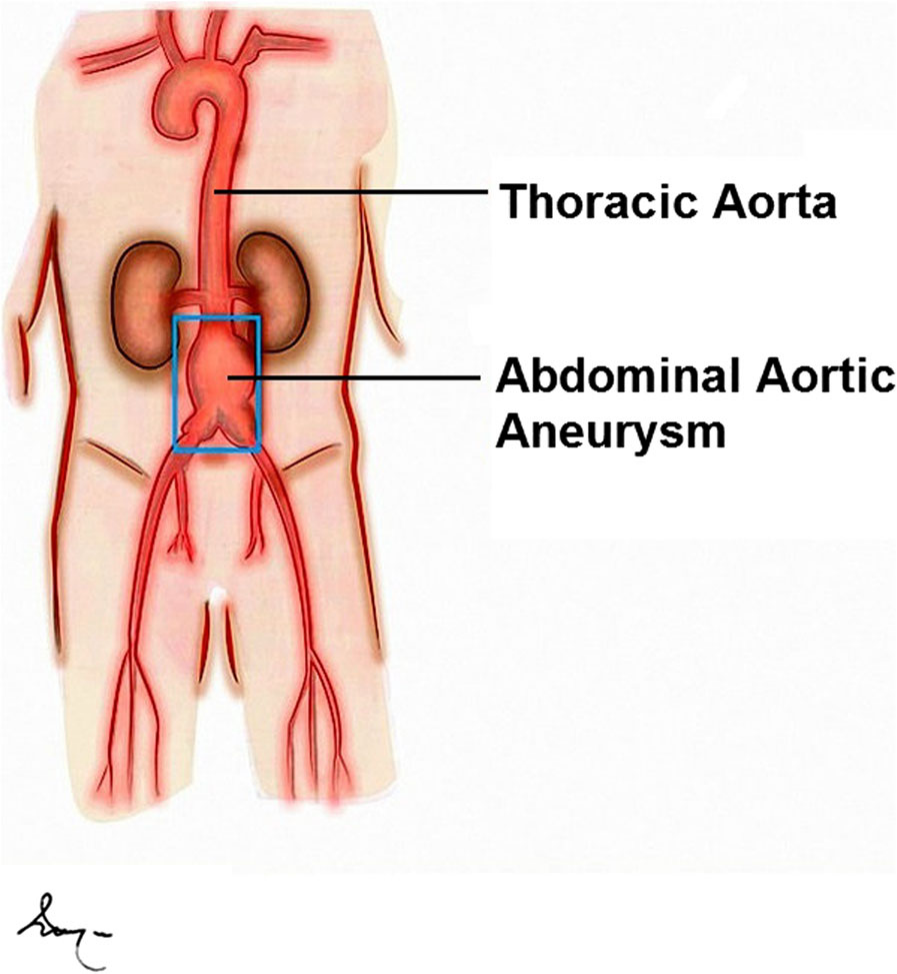
Schematic drawing of the human aorta and the location in the infrarenal portion of an Abdominal Aortic Aneurysm (AAA)

Abdominal Aortic Aneurysms (AAA) are defined as focal dilations located in the abdominal aorta from a diameter of at least 50% greater than the normal and healthy segment of this blood vessel (Fig. 1) [1, 2]. The most common location of this disease process is the infrarenal aorta where a diameter that is equal or greater than 30 mm measured by Computed Tomography Angiography (CTA) establishes the diagnosis of AAA [1, 2], Aortic aneurysms of atherosclerotic or degenerative etiology are relatively frequent among men over 65-year-old with a reported prevalence ranging from 3 to 7% determined by imaging studies [1–3]; and its most feared complication is the aneurysm rupture with an associated mortality above 80%. In the United States of America (USA), this entity constitutes the 13th *leading* overall cause of *death* and according to some authors, this figure is likely to be an underestimation due to approximately 5% of people who die from sudden death may have a rupture AAA as the cause [4]. The associated risk factors for the development of this disease include family background, male gender, dyslipidemia, arterial hypertension and chronic obstructive pulmonary disease (COPD); smoking is a recognized major environmental factor that may enhance the development and the possibility for rupture [3, 4].

The pathogenesis of this type of AAA is recognized to be multifactorial; however, previous genetic studies have suggested the possible roles played by autoimmunity, genetic predisposition and ethnic susceptibility [5, 6], In regard to the ethnicity, the Life Line Ultrasound Screening program performed in the USA from 2003 to 2008 with a sample of 23,466 patients with AAA indicated that African Americans, Asians and Hispanics had a lower prevalence than individuals from European ancestry [5, 7], The mechanism of ethnic susceptibility remains not well understood, and it is difficult to consider particularly the “Hispanic” population as a single ethnic group because they are genetically diverse, being predominantly “Mestizos” as a result of the miscegenation of the Native Americans with Europeans since the sixteenth century and the subsequent arrival of African populations the following centuries to certain regions of the New World[7, 8],

In our institution which is located in Mexico City, the implementation of imaging screening programs has detected a prevalence of 3.26% for abdominal aortic aneurysms in patients at the age of 65 or older suggesting that aortic aneurysmal disease in Mexico is not as uncommon as previously thought [8, 9], In this matter, population genetics studies of Class II Human Leukocyte Antigens in our institution have shown that Mexican mixed-ancestry populations have complex genetic structures with contributions from Native Americans (50–60%), Europeans (25–40%), African (4–12%) and more recently from Asian roots (1%) [10, 11].

To further clarify possible associations of risk of developing AAA between HLA the Class II Human Leukocyte Antigens and particularly the HLA-DRB1 loci in the Mexican Mestizo population, we investigated alleles in patients with and without atherosclerotic aortic aneurysms.

## Methods

**Fig. 2:**
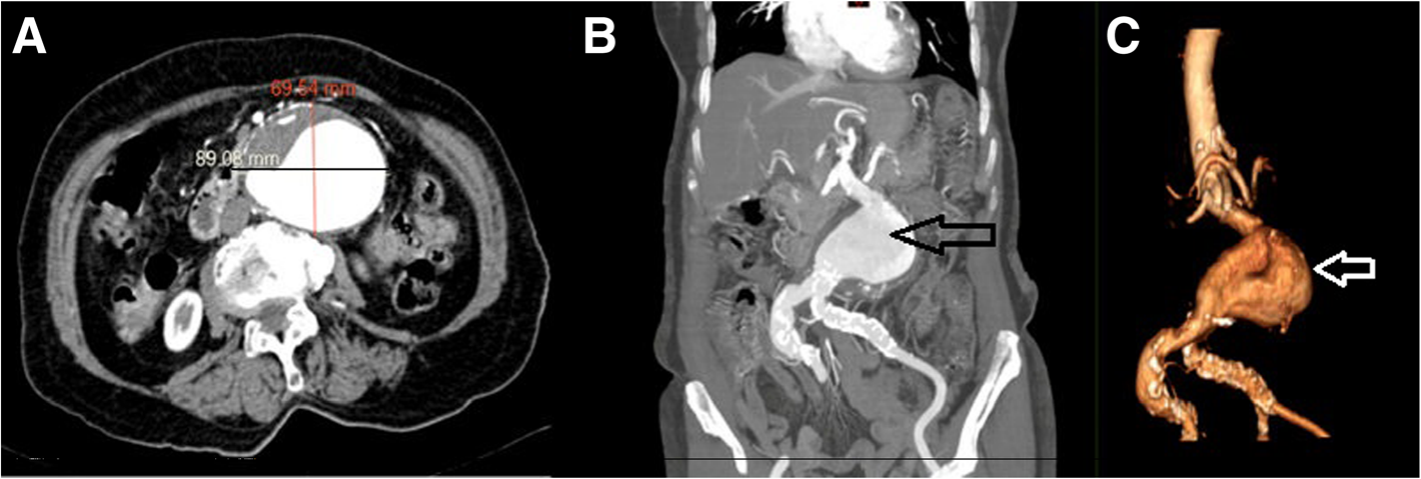
Contrast Tomography Angiography (CTA) in a 66 year old woman. Axial view (**a**) demonstrates a maximum diameter of 89 mm (mm) and antero-posterior diameter of 69 mm. Coronal View (**b**), and three dimensional (3D) reconstruction (**c**) black and white arrows indicate the aneurysm

Case Control Study was performed, HLA molecular typing was completed for DRB1 loci by LabType Sequence-Specific Oligonucleotide (SSO) SSO-OneLambda kit (Applied Biosystems; Thermo Fisher Scientific. Inc.) in the studied case and control groups. Eligible patients included those individuals born in Mexico whose parents and grandparents were also born in Mexico with and without the disease (Fig. 2a-c). Patients with aneurysms associated or secondary to connective tissue disorders, vasculitis, trauma, infectious etiology were not included; the control group was paired and matched in age, gender and ethnicity and aneurysmal disease was ruled out [8, 9], This study was approved by the Institutional Review Board and Ethics Committee at the National Institute of Medical Sciences and Nutrition Salvador Zubiran with the number 1913, informed consent was obtained from patients, including consent to publish. Patient’s data is protected and anonymous.

### Statistical analysis

The frequency of HLA-DRB1 alleles [allele frequencies (af)] was calculated and data were analyzed. We used the Bonferroni method to reduce the possibility of type I errors (multiplying by 10), and *p* values < 0.05 were considered significant. Odds Ratios (OR), which reflect the likelihood of a subject carrying a specific allele, and the 95% confidence interval (95% CI) were calculated. Known alleles that were significantly associated with disease outcome differences were tested by nonparametric statistics as Chi square and fisher exact tests (two tailed) using the STATA Software version 14 and StatCalc software *Epi Info*™ 7.2.2.2. The studied population fits Hardy-Weinberg equilibrium.

### Results

The genetic polymorphism of HLA-DRB1 of 51 patients (70% males, with a mean age of 71 years) with atherosclerotic or also known as degenerative AAA was compared with 99 unrelated patients (60% males, with a mean age of 65 years) without the disease who had no family history of AAA, providing 198 alleles [Control group (CG)] from the same ethnic and age group. A total of one hundred and two alleles of AAA patients and 198 from the CG were examined. When comparing af, we observed the HLA-DRB1*01 af of 0.139 in the AAA compared to 0.05 in the CG [*p* = 0.015, OR 3, 95% confidence interval (CI) 1.29–7.08], the HLA-DRB1*16 af were 0.109 in the AAA and 0.025 in CG (*p* = 0.006, OR 4.7, 95% CI 1.59–13.98). Table 1 summarizes the AAA and CG patients’ demographics and comorbidities; no statistical differences were found in age, gender and smoking history. Table 2 summarizes the af comparison the AAA patients and CG, including *p* value, Odds Ratios (OR) and Confident Intervals (CI). Bonferroni correction was utilized to adjust the *p* values.

**Table 1:** Demographics and comorbidities of Abdominal Aortic Aneurysms and control group (CG) patients

Variable	Number of AAA patients 51 (100%)	Number of CG patients 99 (100%)
Males	36 (70%)	59 (60%)
Females	15 (30%)	40 (40%)
Smoking history	36 (70%)	61 (62%)
Comorbidities		
Arterial Hypertension	35 (69%)	22 (22%)
Dyslipidemia	22 (43%)	15 (15%)
Type 2 Diabetes Mellitus	11 (21%)	9 (9%)
Coronary Artery Disease	11 (21%)	5 (5%)
Peripheral Arterial Disease or Cerebrovascular Accident	7 (14%)	3 (3%)

**Table 2:** Allele frequencies of HLA-DRB1 locus in Mexicans Mestizo patients with Abdominal Aortic Aneurysm (AAA) and control group (CG)

HLA	Cases (AAA) 102 Alleles		Control Group CG 198 Alleles				
	Number of Alleles	Allele Frequency	Number of Alleles	Allele Frequency	*P* Value	Odds Ratio (OR)	Confidence Intervals (CI)
DRB1*04	23	0.228	47	0.237	0.966	0.9	0.54–1.67
DRB1*01	14	0.139	10	0.05	0.015*	3	1.29–7.08
DRB1*16	11	0.109	5	0.025	0.006*	4.7	1.59–13.98
DRB1*07	9	0.089	22	0.111	0.697	0.7	0.35–1.77
DRB1*11	8	0.079	20	0.100	0.669	0.7	0.32–1.78
DRB1*15	9	0.089	13	0.065	0.617	1.3	0.57–3-38
DRB1*14	8	0.079	21	0.105	0.592	0.7	0.31–1.70
DRB1*08	9	0.089	33	0.165	0.099	0.5	0.22–1.07
DRB1*13	4	0.040	10	0.050	0.895	0.8	0.24–2.54
DRB1*03	4	0.040	11	0.055	0.751	0.7	0.22–2.26
DRB1*10	1	0.010	1	0.005	1.000	1.9	0.12–31.83
DRB1*09	1	0.010	3	0.015	1.000	0.6	0.07–6.83
DRB1*12	1	0.010	2	0.010	1.000	0.9	0.09–10.94

* Statistical Significance

## Discussion

The development of an aneurysm in the abdominal aorta seems to be the interaction of environmental and genetic factors [1, 2]. The pathophysiology of this disease process consists in the degradation of the extracellular matrix of the blood vessel wall in response to the accumulation of inflammatory cells such as macrophages and lymphocytes, and the activation of matrix metalloproteinases; and the resulting inflammation, tissue destruction and necrosis produce more cycles of inflammation [2]. The elevation of cytokine levels and the titers of immunoglobulin, both extracted from nonreactive diseased aortic tissue has led to researchers to propose a possible role played by autoimmunity similar to the cases of systemic vasculitis that affect great vessels [2, 11–13]. The Human Leukocyte Antigens (HLA) is an integral component of the immune response on which few genetic studies concentrated in the 1990s and early twenty-first century [14–20] different studies of alleles in various ethnic groups of the locus HLA-DRB1 demonstrated playing a role in the development of AAA [14–20], while one did not demonstrate this association [20]. (Table 3) It is well known that HLA genes are highly polymorphic and that the allele distribution varies in each ethnic group, and it is also known that HLA alleles or polymorphisms of genes within the HLA region are associated with inflammatory arterial disease suggesting a possible autoimmune etiology as it occurs in Takayasu’s Arteritis (TA). The frequency of specific block combination between major ethnic groups of different continental ancestries and these variations can be used as a measurement of MHC genetic diversity in autoimmune conditions, but it is clear that the association seems not universal for all investigated populations [15–20, 22]. As aforementioned, Mexico was colonized primarily by the Spaniards, who arrived to the new world in the early sixteenth century and following the conquest an important admixture between these populations was carried out providing susceptibility and protection to certain diseases [11, 22]. The process in which HLA might confer susceptibility is not fully understood and it is possible that there must be a breakdown of the immunoregulatory mechanisms or molecular mimicry following certain pathogens exposure. We studied the HLA-DRB1 locus because the main products of these proteins present autoantigens to CD4 cells and, as a result of antigen presentation, these cells cooperate with B cells in the release of autoantibody products. We may hypothesize that the pathogenesis of AAA might share similarities with TA, a systemic vasculitis that affects mainly the aorta and its branches and degenerate in stenotic lesions and aneurysm formation. In the case of TA, authors have demonstrated the proliferation of TCD3 lymphocytes against heat shock proteins (HSP) of mycobacterium HSP65 and its human homologue HSP60, and the presence of antibodies IgG against both proteins, suggesting previous infections, possibly as a result of shared epitopes of HSPs leading to autoimmunity [23]. Interestingly one of the alleles of susceptibility that we found in our study, the HLA-DRB1*16 has been also found to be associated with the susceptibility for TA in Colombian Mestizos patients, suggesting a possible similar mechanism for atherosclerotic aneurysms that requires further investigation [24]. We agree that the pathogenesis of aortic aneurysmal disease is clearly multifactorial, however the genetic factors seem important, and possibly the result of the interaction and contribution of other genes and environmental factors [5, 6].

**Table 3:** Summary of case control studies of the association of HLA-DRB1 and Abdominal Aortic Aneurysms

	Author	Year of Publication	HLA Isotypes and Subtypes	Cases/Controls	P Value	Odds Ratio OR	95% CI
1	Tilson [6]	1996	HLA-DRB1*02		*P* = 0.037	NR	NR
			HLA-DRB1*12*	5/NR	*P* = 0.023		
2	Rasmussen [21]	1997	HLA-DRB1*15	37/90	*p* < 0.05	NR	NR
			HLA-DRB1*0404				
3	Hiroshe [15]	1998	HLA-DRB1*2 (15)	46/50	*P* < 0.005	NR	NR
4	Rasmussen [16]	2001	HLA-DRB1*02,	102/118	*P* = .03	2.2	1.2–4.0;
			HLA-DRB1*04	Degenerative	*P* = .01	2.0	1.1–3.7,
			HLA-DRB1*02,	40/118		3.7	1.8–8.6;
			HLA-DRB1*04	Immflamatory		2.5	1.1–6.1
5	Rasmussen [17]	2002	HLA-DRB1*02,	96/NR	NR	2.5	1.4 a 4.3
			HLA-DRB1*04			2.1	1.2 a 3.7
6	Monux [18]	2003	HLA-DRB1*04	72/380	*P* = 0.02	2.59	1.4 a 4.3
7	Sugimoto [19]	2003	HLA-DRB1*1502.	46/40	p < 0.005	NR	NR
8	Badger [20]	2007	HLA-DRB1*03, *04, *07, and *15	241/NR	NS	NR	NR

*NR* Not reported

**Fig. 3:**
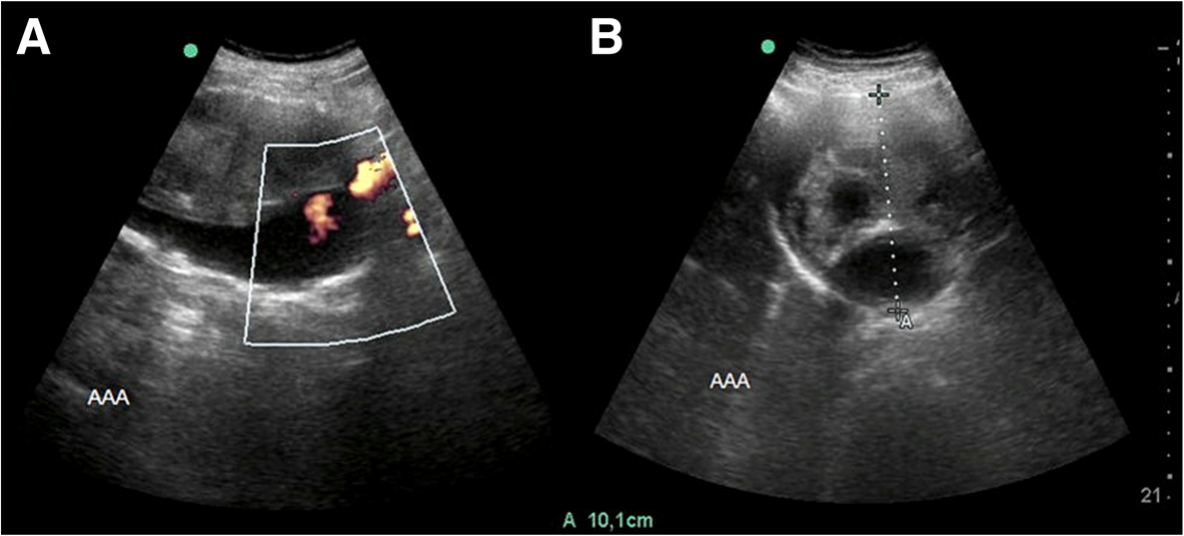
**a, b**. Ultrasonographic study in a 65 year old male patient with an Abdominal Aortic Aneurysm (AAA) (A), with a maximum diameter of 10.1 cm (cm) determined by this imaging study. *Permission to reproduce granted by Permanyer Hinojosa CA, Bermudez-Serrato K, Anaya-Ayala JE,* et al. *Proactive measurements in the search of aortic aneurysms have an impact in the prevalence. Cir Cir. Article in press*

Abdominal Aortic Aneurysms are typically asymptomatic and early detection represent a fundamental step to eliminate the risk of rupture. In the most recent Clinical Practice Guidelines from the Society for Vascular Surgery (SVS) in USA, the expert panel recommend that patients at the age of 65 or greater with a smoking history enter an ultrasound (US) screening program with the objective of identifying asymptomatic aneurysms providing timely elective surgical or endovascular treatment to avoid potentially lethal complications such as rupture (See Fig. 3a, b) [25, 26]. Presently, the evaluation and follow up of AAA relies only on serial measurements of aortic diameters, which remains a predictor of aneurysms growing and possible rupture. Screening criteria is based primarily on demographic characteristics and patient’s risk factors, and we are convinced that further study into the genomics of AAA development might assist to better determine patients should be studied and followed [25–27]. Our results demonstrate significant association of the alleles HLA-DRB1*01 and HLA-DRB1*16 in the Mexican Mestizo Population with susceptibility to develop aneurysms compared to an age, gender and ethnically matched control group, nevertheless the small sample size of this study suggests further confirmation in a larger cohort of patients and in different genetic populations. We observed and recognized differences in comorbidities among our studied groups that were not controlled during selection process, however the SVS 2018 Clinical Practice Guidelines emphasize that age, gender and smoking history are the most significant risk factors [25]. The results suggest as previous reports the possible role of HLA-DRB1 locus in the pathogenesis of AAA study and based on these findings it might be useful to study an immugenetic profile along with ultrasonographic studies in order to determine the susceptibility to developed AAA. The clinical impact of this technique has not been previously assessed and would require a large-scale clinical study; in this context, we consider that the identification of specific HLA alleles in different populations will be helpful in understanding the genetic background related to this particular condition and establishing optimal and reproducible preventive measurements.

The recognized study limitations include that this is a single-center study performed in a tertiary referral center, and that only low-resolution HLA tests were performed in a small sample from Mexico City and metropolitan areas, making adjustment for potential confounders and covariates in the studied populations including a survival bias. Additionally, our results are based in a single locus analysis and no haplotype data was included in this study, so the contribution of neighboring genes was not evaluated. We are convinced that polymorphic ongoing family studies will provide more information in linked-disequilibrium disease association.

## Conclusions

Our study confirmed increased frequencies of the alleles HLA-DRB1*01 and HLA-DRB1*16 and their association to the development of AAA in Mexican Mestizo patients. The utility of genetic testing may assist in identifying individuals at genetic risk for the development of this disease in different ethnic groups, who might benefit from earlier ultrasound screening and closer imaging surveillance.

## Data Availability

The databases used and/or analyzed during the current study are available from the corresponding author on reasonable request. Confidential patient data are not shared.

## Abbreviations

AAA: Abdominal Aortic Aneurysm
af: Allele frequencies
CG: Control group
HLA: Human Leukocyte Antigens
HSP: Heat shock proteins
SSO: Sequence-specific Oligonucleotide
TA: Takayasu’s Arteritis
